# Mindfulness-Based Smoking Cessation Enhanced With Mobile Technology (iQuit Mindfully): Pilot Randomized Controlled Trial

**DOI:** 10.2196/13059

**Published:** 2019-06-24

**Authors:** Claire Adams Spears, Lorien C Abroms, Carol R Glass, Donald Hedeker, Michael P Eriksen, Cherell Cottrell-Daniels, Binh Q Tran, David W Wetter

**Affiliations:** 1 Department of Health Policy and Behavioral Sciences Georgia State University School of Public Health Atlanta, GA United States; 2 Prevention and Community Health Milken Institute School of Public Health George Washington University Washington, DC United States; 3 Department of Psychology The Catholic University of America Washington, DC United States; 4 Department of Public Health Sciences The University of Chicago Chicago, IL United States; 5 Department of Biomedical Engineering The Catholic University of America Washington, DC United States; 6 Center for Health Outcomes and Population Equity University of Utah and Huntsman Cancer Institute Salt Lake City, UT United States

**Keywords:** text messaging, smoking cessation, low-income populations

## Abstract

**Background:**

Mindfulness training shows promise for improving smoking cessation and lapse recovery, and between-session mobile health messages could enhance treatment engagement and effectiveness. Personalized, in-the-moment text messaging support could be particularly useful for low-income smokers with fewer smoking cessation resources.

**Objective:**

This pilot study examined the feasibility of a text messaging program (*iQuit Mindfully*) as an adjunct to in-person Mindfulness-Based Addiction Treatment (MBAT) for smoking cessation.

**Methods:**

A total of 71 participants were randomly assigned to MBAT (n=33) or iQuit Mindfully (n=38; MBAT + between-session text messages); of these, 70% (50/71) were African American, and 61% (43/71) had an annual household income of US $30,000 or less. All participants received 8 weekly therapist-led group counseling sessions, nicotine patches, and self-help materials. Outcomes were feasibility (attrition, engagement, and participants’ ratings), participants’ feedback regarding the text messaging intervention, and smoking cessation (assessed in person).

**Results:**

Strong retention was achieved (76% [54/71] at the end of treatment, and 89% [63/71] at 1-month follow-up). In the iQuit Mindfully group, engagement was high (88% [29/33] indicated reading all or most texts, and 89% [34/38] engaged in interactive texting), and participants provided positive ratings (on a 1-10 scale, average rating for recommending the program to others was 8.4 [SD 2.5]). Participants indicated benefiting from the texts (eg, appreciating encouraging reminders, coping strategies, and social support) and suggested improvements (eg, more personalization). Overall, biochemically confirmed smoking cessation rates were 22% (12/55) at the end of treatment and 19% (12/62) at 1-month follow-up, with no differences between conditions. Living below the poverty level predicted worse cessation outcomes at 1-month follow-up among participants receiving in-person only treatment (*P*=.03) but not among those receiving iQuit Mindfully.

**Conclusions:**

Text messaging appears to be a feasible and acceptable modality for supporting mindfulness-based smoking cessation treatment. The availability of 24/7 text messaging might be particularly helpful for low-income smokers who have access to fewer cessation resources and experience significant day-to-day barriers to quitting.

**Trial Registration:**

ClinicalTrials.gov NCT03029819; https://clinicaltrials.gov/ct2/show/NCT03029819

## Introduction

### Background

Smoking is the leading cause of premature death in the United States [1]. Only 7% of smokers quit each year despite most indicating an interest in quitting [2], and profound tobacco-related health disparities exist [3]. Smoking prevalence remains disproportionately high among adults with low socioeconomic status (SES) despite substantial decline in the smoking rate in the general US population [3-5]. One-fourth (25.3%) of adults living below the federal poverty line smoke, compared with only 14.3% of those at or above the poverty level [5]. Low-SES smokers and members of certain racial and ethnic minority groups, including African Americans, often have greater difficulty quitting and have higher incidence and mortality rates for tobacco-related cancers [2,3,6,7]. Smokers with poorer financial, structural, and social resources face formidable day-to-day barriers, including societal (eg, low health care access), community (eg, tobacco advertising and neighborhood stress), interpersonal (eg, social norms for smoking and low social support), and intrapersonal factors (eg, high stress and low self-efficacy) [3,6,8], which promote addiction and impede efforts to quit. Improving on evidence-based smoking cessation interventions for low-SES and racial and ethnic minority populations will be critical for targeting tobacco-related health disparities.

### Mindfulness-Based Programs for Smoking Cessation

Training in mindfulness (ie, purposeful, nonjudgmental, present-focused attention [9,10]) shows promise for increasing rates of smoking cessation and lapse recovery [11,12]. Mindfulness refers to one’s relationship with his or her thoughts and emotions (ie, observing these experiences nonjudgmentally, without reacting or trying to change them) rather than to their content. A meta-analysis of randomized controlled trials (RCTs) found that participants receiving mindfulness interventions were almost twice as likely to achieve smoking abstinence for more than 4 months compared with those receiving usual care (25.2% vs 13.6%) [13]. There is a dearth of research on mindfulness in low-SES and racial and ethnic minority groups, but mindfulness does show promise for smoking cessation in these populations [14,15]. Programs that teach nonjudgmental, self-compassionate awareness could be particularly useful for racial and ethnic minority populations [16] and have been perceived as empowering among low-SES and racial minority adults [17]. However, additional between-session support may be needed for low-SES and racial and ethnic minority smokers, who experience significant day-to-day barriers to quitting and have lower access to smoking cessation resources.

### Mobile Health and Smoking Cessation

Mobile health (mHealth) interventions have promise for encouraging skills on a real-time, real-life basis, thus increasing skill level, self-efficacy, and the likelihood that the skill will become a part of daily routines [18]. mHealth messages could encourage participants to use mindfulness and other smoking cessation strategies in moments of high stress or craving. This type of in-the-moment support could be especially beneficial for populations (eg, low-SES smokers) with fewer cessation resources. Recent research supports the promise of technology (eg, Web-based training and mobile apps) for teaching mindfulness [19,20], including for smoking cessation [21-23]. Although mindfulness apps have been proliferating, most mHealth mindfulness programs have not been rigorously tested [24].

There is strong empirical support for text messaging programs for smoking cessation [25-30], although none to our knowledge has focused on mindfulness. In a systematic review, mobile phone interventions (most using text messaging) increased smoking abstinence at 6 months (risk ratio [RR]=1.67), with even more positive findings for biochemically verified abstinence (RR=1.83) [26]. Text messaging does not require a smartphone, internet access, or high technical literacy, thus meeting the needs of many adults with lower SES. For example, the vast majority (91%) of college graduates own a smartphone compared with only 57% of adults with less than high school education [31]. However, 90% of Americans with less than high school education own a mobile phone [31]. Furthermore, low-SES and certain racial and ethnic minority adults use text messaging particularly often. In a Pew Research Center study, mean number of texts sent/received per day for Caucasians, African Americans, and Latinos were 31.2 (median 10), 70.1 (median 20), and 48.9 (median 20), respectively. Whereas mean texts per day among adults with college education or greater was 23.8 (median 10), those with less than high school education sent/received 69.4 texts per day (median 20) [32]. Text messaging can provide strategies and encouragement in the context of everyday life and in real time (eg, in moments of high stress or craving), and the content of messages can be personalized. As an adjunct to in-person mindfulness treatment, between-session text messaging could increase treatment engagement and provide vital 24/7 support for smokers from disadvantaged backgrounds.

### iQuit Mindfully

Recognizing that most mHealth programs have been developed without adequate feedback from the target population [33], we took a user-centered design approach [34,35] to develop a text messaging smoking cessation program for predominantly low-SES, racially/ethnically diverse smokers. As described in detail elsewhere [36], we conducted 2 phases of formative research (initial focus groups before developing text message content, and then an abbreviated 1-week trial of text messages) to gather qualitative data to inform and improve the text messaging program. User feedback was elicited throughout the process of developing and refining the messages. The text messages were designed to be sent between weekly in-person mindfulness treatment sessions for smoking cessation.

This study is a pilot investigation of mindfulness-based smoking cessation that incorporates this between-session text messaging (*iQuit Mindfully*). To our knowledge, this is the first study to use text messaging to enhance mindfulness-based smoking cessation treatment. Participants were randomly assigned to 1 of 2 groups: Mindfulness-Based Addiction Treatment (MBAT) or iQuit Mindfully (MBAT with the addition of between-session text messages). Feasibility and acceptability outcomes critical to this pilot study were attrition, participant engagement with text messages, and participant ratings and feedback regarding the text messaging program. The primary smoking cessation outcome was 7 consecutive days of abstinence from smoking at the end of treatment. In addition, secondary analyses examined associations among engagement with text messaging, mindfulness practice, and smoking cessation, as well as cessation outcomes by poverty status.

## Methods

### Participants

Recruitment targeted a racially/ethnically diverse sample of smokers with relatively low income levels in the Atlanta, GA, area. Inclusion criteria were age 18 to 65 years; current smoker with history of ≥5 cigarettes per day for the past year (and expired carbon monoxide [CO] ≥6 ppm); motivated to quit within the next 30 days; valid home address in the greater Atlanta area; functioning telephone number; owning a mobile phone with text messaging capacity; ability to speak, read, and write in English; and marginal/adequate health literacy (at least a sixth grade level) as determined by the Rapid Estimate of Adult Literacy in Medicine [37]. Exclusion criteria were contraindication for the nicotine patch; past 30-day use of recreational drugs, alcohol-related problems (positive response on 2 or more of the 5 Patient Health Questionnaire [PHQ] Alcohol Abuse/Dependence Scale items [38]), self-reported current diagnosis of schizophrenia or bipolar disorder or use of antipsychotic medications, score of ≥3 on the PHQ-2 [39] depression screening instrument, regular use of tobacco products other than cigarettes (electronic cigarette users were not excluded), current use of tobacco cessation medications, pregnancy or lactation, or another household member enrolled in the study. The study was approved by the university’s institutional review board, and written informed consent was obtained from all participants.

### Procedures

Participants were recruited through flyers (posted at venues including the university’s downtown campus, local hospitals/community health centers, and near bus and train stops), Web-based sources (eg, Craigslist, listservs), and word of mouth. After screening and informed consent procedures, participants were randomly assigned to iQuit Mindfully (MBAT with text messaging) or MBAT (without text messaging). Randomization took place at the end of the baseline session, after baseline assessments had been administered. Permuted block randomization, with stratification based on age (ages 18-49 vs 50-65 years), was used to allocate participants to treatment condition. Co-author DH used SAS software (SAS Institute Inc) to generate the random number sequence. A graduate research assistant (unaware of the size of the blocks) allocated interventions through opaque sealed envelopes marked according to the allocation schedule. The majority of study personnel were masked to treatment condition. Limited staff were unmasked to handle randomization codes (ie, the graduate research assistant) and delivery of interventions (ie, the study therapist). Participants were recruited between January and June 2017; interventions were delivered between February and September 2017; and follow-up assessments were conducted between May and October 2017.

### Study Interventions

All participants received in-person group treatment based on the 8-week MBAT protocol [12], in addition to the 6 weeks of nicotine patch therapy and self-help materials based on the Treating Tobacco Use and Dependence Clinical Practice Guideline [40]. Patch therapy (beginning on the quit day) for participants who smoked more than 10 cigarettes per day consisted of 4 weeks of 21 mg patches, 1 week of 14 mg patches, and 1 week of 7 mg patches. Patch therapy for those who smoked 5 to 10 cigarettes per day consisted of 4 weeks of 14 mg patches and 2 weeks of 7 mg patches.

MBAT was provided by a master’s level licensed professional counselor with formal training in mindfulness and addictions. MBAT closely follows Mindfulness-Based Cognitive Therapy [41] procedures but replaces the depression-related material with nicotine dependence–related material. The program consists of 8 weekly 2-hour sessions. Aims are to help participants increase moment-to-moment awareness of thoughts, feelings, and sensations; observe these sensations nonjudgmentally; and learn to disengage their attention and choose more skillful responses (rather than automatic reactions) to uncomfortable sensations (including cravings) and high-risk situations. To provide additional support on the quit date and encourage further mindfulness practice, session 5 (quit date) was an extended 4-hour session. MBAT emphasizes daily practice in several forms: formal sitting meditation, body scan meditation, walking meditation, eating meditation, and gentle yoga. There were between 8 and 15 participants in the MBAT group sessions.

Participants in the iQuit Mindfully condition also received approximately 2 to 6 text messages per day on each day between treatment sessions. Texts were sent using the Mobile Commons/Upland Mobile Messaging platform. The content and frequency of messages were revised based on focus groups and pilot testing with low-income, racially/ethnically diverse smokers [36]. Text messages reminded participants to practice mindfulness (eg, reminders for informal practice, such as awareness of the breath throughout the day, and reminders for formal practice, such as the body scan and sitting meditation). Text messages were personalized (eg, reminding participants of their personal reasons to quit and amount of money to be saved as a result of quitting; incorporating first names) and interactive (eg, participants were asked questions such as “Good morning, John! Would you like to try a mindfulness exercise?” and “There are people, places, and things that make you want to smoke. What are your top 3 triggers to smoke?”), and automated text responses were sent based on their replies. Texts also provided specific strategies to aid in cessation (eg, reminders to get rid of cues to smoke, reach out for social support, and coping strategies taught in MBAT).

Participants received approximately 2 messages per day during week 1, 3 per day during week 2, 4 per day during week 3, 5 per day during week 4, 6 per day during week 5, 4 per day during week 6, and 3 per day during week 7. This message schedule was based on our earlier qualitative work [36]. Participants could also text specified words (CRAVE, STRESS, or SLIP) at any point to receive additional text message support for coping with cravings, stress, or smoking lapses, respectively. Participants received a relatively small number of texts (1-3 per week) during the 1-month follow-up period and had the opportunity to text the CRAVE/STRESS/SLIP keywords during this time.

### Measures

#### Demographics and Baseline Smoking Behavior

At baseline, participants indicated their gender, age, education, income, and employment status. Poverty status (below vs at or above the federal poverty line) was calculated according to US Census Bureau Guidelines based on family size and number of children [42]. The Heaviness of Smoking Index, a strong indicator of nicotine dependence [43], assessed self-reported average number of cigarettes smoked per day and the time to first cigarette on waking.

#### Participants’ Ratings and Feedback Regarding iQuit Mindfully

Participants in the iQuit Mindfully condition completed program evaluation forms in person at the end of treatment. Participants were asked, “Of all of the text messages that you received as part of this program, how many did you read?” (response options: *none*, *some*, *most*, or *all*). They were also asked, “Overall, how helpful were the text messages in getting you to try to quit smoking?” (rated from 1=not at all helpful to 10=extremely helpful), and then specifically asked about the helpfulness of the CRAVE, STRESS, and SLIP keywords using the same 10-point scales. They were prompted to “Please circle the number that best represents whether you would recommend that other people receive the text messages that you received in this program (or similar texts) as a way to help them quit smoking” (rated from 1=would not recommend to 10=would definitely recommend). It was determined a priori that scores of 6 or more on these 10-point scales would indicate that the texts were acceptable. Participants were also asked, “Please rate the overall *number* of text messages that you received as part of this program”: (response options: “not enough texts,” “prefer more texts,” “about the right number of texts,” “prefer fewer texts,” and “way too many texts”).

Finally, participants were asked the following open-ended questions:

- What did you like the most about the text messages?
- How, if at all, did you find the text messages to be helpful? (these 2 items were combined for analysis given considerable overlap)
- What, if anything, did you dislike about the text messages?
- What recommendations do you have to improve the text messages?

#### Smoking Abstinence

Smoking abstinence at the end of treatment (3 weeks after quit date) and 1-month follow-up (7 weeks after quit date) was defined as self-reported complete abstinence for 7 days that was biochemically confirmed in person with CO <6 ppm. Participants who denied smoking for the past 7 days but had CO levels ≥6 ppm were coded as not abstinent (n=4 at the end of treatment and n=2 at follow-up). Missing data were not coded as smoking because of the potential for severe bias that has been demonstrated in prior studies [44,45].

#### Weekly Mindfulness Practice

Each week from treatment session 2 to session 8, participants completed a weekly mindfulness practice log [12] to indicate the number of days that they practiced each of 5 core mindfulness techniques taught in treatment (sitting meditation, body scan, walking meditation, yoga/mindful stretching, and mindful awareness of breath during the day) during the past week. Responses were averaged for each mindfulness practice over the course of treatment.

### Data Analysis

Descriptive statistics provided information on feasibility and acceptability (ie, attrition, engagement, and participants’ ratings). Open-ended responses to program evaluations were coded using QSR International’s NVivo 11 software. The first author (CAS) and coauthor CCD each reviewed the responses to develop an initial set of themes and then collaborated to define specific codes and refine the coding manual. CAS and CCD then each separately coded all the responses, with an overall kappa of .95, indicating high interrater reliability. Discrepancies were resolved through discussion, with final decisions made by the first author. Chi-square tests examined group differences in smoking cessation outcomes at the end of treatment and 1-month follow-up. Independent samples *t* tests examined group differences in mindfulness practice over the course of treatment.

In addition, 2 ancillary analyses were conducted. First, to examine whether participants who were more engaged with the text messages and/or practiced mindfulness more frequently between sessions had better outcomes, associations among text message engagement (based on the number of times participants texted the system), weekly mindfulness practice variables, and abstinence were examined using chi-square, *t* tests, and logistic regression. Second, because the text messages were specifically designed to target low-SES smokers, analyses examined results separately by poverty status (below vs at or above the federal poverty line).

## Results

### Screening and Enrollment

**Figure 1:**
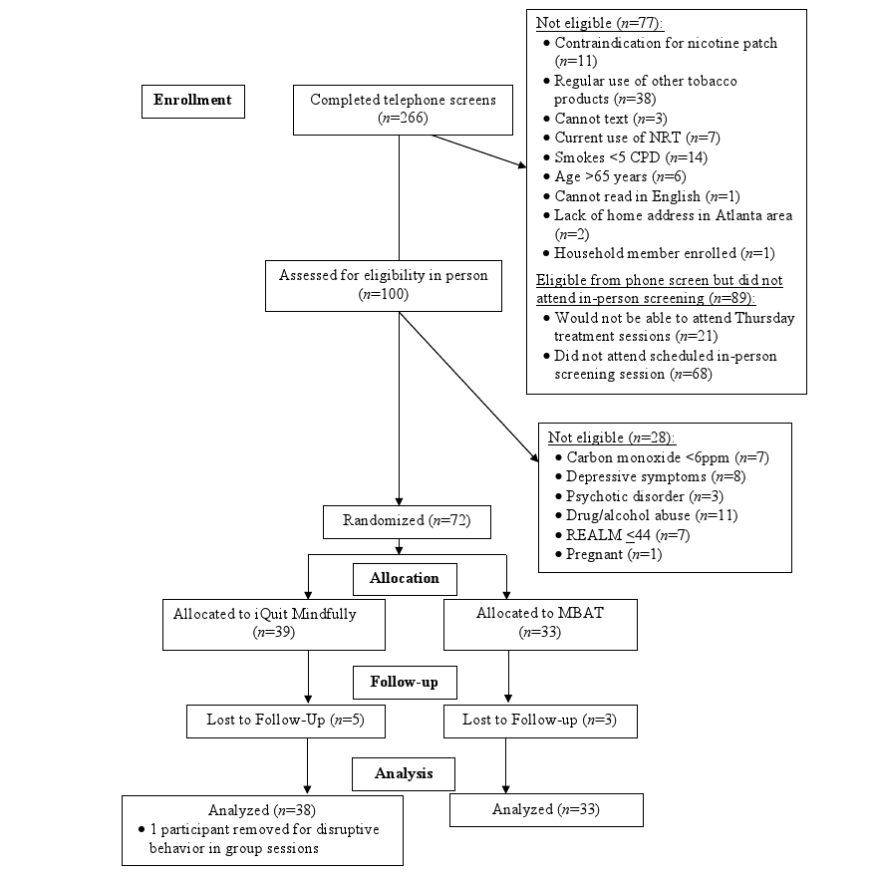
Consolidated Standards of Reporting Trials flow diagram. CPD: cigarettes per day; MBAT: Mindfulness-Based Addiction Treatment; NRT: nicotine replacement therapy; REALM: Rapid Estimate of Adult Literacy in Medicine.

A total of 266 individuals completed telephone screening, 100 completed in-person screening, and 72 participants were enrolled in the study (Figure 1). Overall, 1 participant in the iQuit Mindfully condition was removed because of disruptive behavior in the in-person group treatment for a final analytic sample of 71.

### Participants’ Characteristics

Demographic and smoking-related characteristics at baseline are shown in Table 1. Mean age of the participants was 45.6 years (SD 12.1), and about half (37/71, 52%) were female. Most participants were African American (50/71, 70%), 15 (21%) were Caucasian, and 4 (6%) reported more than 1 race. Most (43/71, 61%) indicated having an annual household income of US $30,000 or less, and 41% (27/66) were living below the federal poverty line. On average, participants smoked 16.5 (SD 9.6) cigarettes per day at baseline and had been smoking daily for 23.6 (SD 14.1) years. The majority (57/71, 80%) reported smoking their first cigarette within 30 min of waking, and most (57/71, 80%) smoked primarily menthol cigarettes. Although statistical tests were not conducted to examine baseline differences between treatment groups [46,47], examination of the descriptive statistics suggests that participants in the iQuit Mindfully condition were more likely to be African American and have lower SES than those in MBAT.

**Table 1:** Participants’ characteristics.

Demographic characteristics		Full sample (N=71)	iQuit Mindfully (n=38)	Mindfulness-Based Addiction Treatment (n=33)
Age (years), mean (SD)		45.6 (12.1)	45.6 (12.4)	45.6 (12.0)
Gender, female, n (%)		37 (52)	17 (45)	20 (61)
**Race/ethnicity, n (%)**				
	Black/African American	50 (70)	34 (89)	16 (49)
	Caucasian	15 (21)	4 (11)	11 (33)
	Asian	1 (1)	0 (0)	1 (3)
	More than 1 race	4 (6)	0 (0)	4 (12)
	Other	1 (1)	0 (0)	1 (3)
	Hispanic/Latino	3 (4)	1 (3)	2 (6)
**Employment, n (%)**				
	Regular full-time work (40+ hours/week)	17 (24)	6 (16)	11 (33)
	Regular part-time work	13 (18)	3 (8)	10 (30)
	Temporary part-time work	1 (1)	1 (3)	0 (0)
	Self-employed	1 (1)	1 (3)	0 (0)
	Student	4 (6)	2 (5)	2 (6)
	Unemployed	19 (27)	15 (39)	4 (12)
	Retired	10 (14)	5 (13)	5 (15)
	Unable to work or disabled	6 (9)	5 (13)	1 (3)
**Education, n (%)**				
	Less than high school degree	12 (17)	7 (18)	5 (15)
	High school degree or General Education Development	14 (20)	7 (18)	7 (21)
	Some college/technical school	19 (27)	12 (32)	7 (21)
	Associates degree	10 (14)	4 (11)	6 (18)
	Bachelor’s degree	10 (14)	5 (13)	5 (15)
	Some postbac school	3 (4)	3 (8)	0 (0)
	Graduate degree	3 (4)	0 (0)	3 (9)
**Annual household income in US dollars (n=66), n (%)**				
	≤$12,000	20 (30)	12 (36)	8 (24)
	$12,001-$18,000	10 (15)	7 (21)	3 (9)
	$18,001-$30,000	13 (20)	5 (15)	8 (24)
	$30,001-$42,000	7 (11)	2 (6)	5 (15)
	$42,001-$54,000	2 (3)	0 (0)	2 (6)
	$60,001-$84,000	4 (6)	0 (0)	4 (12)
	>$84,000	10 (15)	7 (21)	3 (9)
**Poverty status (n=66), n (%)**				
	Below poverty threshold	27 (41)	16 (48)	11 (33)
	At or above poverty threshold	39 (59)	17 (52)	22 (67)
Cigarettes per day, mean (SD)		16.5 (9.6)	14.4 (9.4)	18.8 (9.3)
Years smoking daily, mean (SD)		23.6 (14.1)	20.7 (13.1)	27.0 (14.6)
**Time to first cigarette, n (%)**				
	Within 5 min	27 (38)	17 (45)	10 (30)
	6-30 min	30 (42)	15 (39)	15 (45)
	31-60 min	7 (10)	3 (8)	4 (12)
	After 60 min	7 (10)	3 (8)	4 (12)
Menthol cigarettes as regular brand, n (%)		57 (80)	33 (87)	24 (73)

### Treatment Attendance

Participants attended an average of 5.7 of 8 treatment sessions (SD 2.7), with no difference between conditions (iQuit Mindfully: 5.9 sessions, SD 2.7; MBAT: 5.4 sessions, SD 2.8), *P*=.50. Greater treatment attendance predicted higher likelihood of smoking cessation at the end of treatment (β=4.05, *P*=.049) and approached significance in predicting cessation at 1-month follow-up (β=1.76, *P*=.058).

### Feasibility and Acceptability

#### Attrition

A priori, it was deemed that 35% attrition would be acceptable (based on 34% attrition in trial of MBAT by Vidrine et al [12]). Overall attrition rates were 24% (17/71) at the end-of-treatment assessment and 11% (8/71) at 1-month follow-up, with no differences between groups (attrition at the end of treatment: iQuit Mindfully 21% [8/38], MBAT 27% [9/33], *P*=.38; attrition at 1-month follow-up: iQuit Mindfully 11% [4/38], MBAT 12% [4/33], *P*=.83).

#### iQuit Mindfully Engagement

Level of engagement was determined based on (1) the proportion of texts that participants indicated reading (we expected that at least 75% would read most or all texts, based on the study by Abroms et al [48]) and (2) responses to interactive text messages (we expected that at least 85% of participants would respond to at least one of the interactive text messages or use the CRAVE, STRESS, or SLIP keywords at least once, based on the study by Heminger et al [49]). These benchmarks were achieved. The majority (88%, 29/33) indicated reading all or most text messages, and 89% (34/38) responded to at least one of the interactive text messages or used the CRAVE, STRESS, or SLIP keywords.

#### Participants’ Ratings and Feedback

On a scale of 1 to 10, participants’ average rating of text helpfulness was 8.0 (SD 2.4). Ratings regarding the helpfulness of the keywords CRAVE, STRESS, and SLIP were 7.8 (SD 0.29), 7.9 (SD 2.7), and 7.8 (SD 2.8), respectively. On a scale of 1 to 10, participants’ average rating of the extent to which they would recommend the text messaging program to others was 8.4 (SD 2.5). Most (58%, 19/33) indicated receiving about the right number of texts, 30% (10/33) preferred fewer, and 12% (4/33) preferred more.

Table 2 shows themes and illustrative quotations from participants’ open-ended responses on iQuit Mindfully program evaluations. Overall, participants reported positive experiences with the text messages (eg, “I loved them; sometimes I read them going to bed instead of smoking”). Almost all (97%, 32/33) provided positive responses when asked what they liked and what was most helpful about the texts. Themes were appreciating the positive tone (n=13), receiving reminders (n=11), benefiting from mindfulness (n=9), perceiving a sense of social support (n=9), perceiving that the messages had good timing (n=6), noting that the messages encouraged self-compassion in the face of smoking lapses (n=6), and receiving specific strategies to cope with cravings and stress (n=5). When asked what they disliked, 61% (20/33) responded *nothing*, *N/A*, or left the question blank. Whereas 9 participants indicated that there were too many text messages or that they were repetitive, 7 indicated wanting more text messages. When asked their suggestions for improvements, 67% (22/33) responded *none*, *N/A*, or left the question blank. Of those who provided feedback, suggestions included connecting participants to additional outside resources (n=5; eg, connect to phone call, emergency resources, one-on-one support), incorporating more personalization (n=3), and including more religion/spirituality in the messages (n=3).

### Smoking Abstinence

There were no significant differences between groups either at the end of treatment (iQuit Mindfully: 26% [8/31]; MBAT: 17% [4/24], *P*=.42) or follow-up (iQuit Mindfully: 16% [5/32]; MBAT: 23% [7/30], *P*=.44).

### Weekly Mindfulness Practice

Mean number of days engaging in each type of mindfulness practice per week ranged from 2.02 (SD 1.72) for yoga to 3.25 (SD 2.11) for mindful awareness of breathing. There were no significant between-group differences, *P*>.59.

### Associations Among Text Engagement, Weekly Mindfulness Practice, and Abstinence

Among iQuit Mindfully participants, the mean number of times participants texted the system was 55.2 (SD 63.1; median 37) and was highly skewed (7 participants texted over 100 times, with 2 of these texting over 200 times). Thus, a dichotomized text engagement variable was created based on the median. Participants categorized as having high engagement had significantly higher abstinence rates at the end of treatment than those with low engagement, χ^2^_1_=7.8, *P*=.005. Whereas 44% (8/18) of participants with high engagement were abstinent at the end of treatment, none of those with low engagement was abstinent. Engagement was not significantly associated with abstinence at 1-month follow-up (7% [1/14] of those with low engagement vs 22% [4/18] of those with high engagement were abstinent, *P*=.24).

**Table 2:** Example quotations from open-ended iQuit Mindfully program evaluation responses.

Themes			Example quotations
**Most helpful aspects**			
	Positive tone	“Positive response was really cool for my confidence” “Positive and uplifting”	
	Reminders	“They became an integral part of your day and served as gentle reminders and encouragement” “They reminded me of my goals and told me why I was choosing to quit smoking”	
	Mindfulness	“Helped me to stay mindful” “Kept me aware” “Stop breathe think”	
	Social support	“I felt that someone cared how I was feeling” “I was able to reach out for support and it was very helpful” “It let me know somebody out there to help me”	
	Good timing	“Sometimes they came right on time. I would start thinking about smoking and here comes that text.” “Every time I thought about smoking I get that text of encouragement to not smoke.”	
	Self-compassion in the context of smoking lapses	“They encouraged me to continue with my journey and don’t worry about the slip up and just start over.” “Made you not beat yourself up about a slip”	
	Strategies for coping with cravings and stress	“If you text CRAVE and actually do what the text message says you will successfully overcome that current craving” “You were given techniques to help overcome the stress”	
**Dislikes**			
	Too many text messages/repetitive	“Came a little too quick sometimes” “Repetitive” “Less texts would be better”	
	Not enough text messages	“[I disliked] when they became less frequent” “Even more would be helpful”	
**Suggestions**			
	Connect to outside resources	“One on one support. Additional resources” “Maybe a call”	
	More personalization	“Really try to find out what best suits each individual” “More intuitive and spontaneous and less generically programmed”	
	Religion/spirituality	“Send Bible verses/scriptures” “More spiritual texts”	

Associations between engagement with text messages and mindfulness practice were also examined. Participants who showed high text engagement reported practicing informal mindfulness more frequently than those with low engagement (high engagement: mean 3.9 [SD 2.0] days vs low engagement: mean 2.0 [SD 1.6] days), *t*_32_=2.91, *P*=.006. The association between text engagement and frequency of sitting meditation practice approached significance (high engagement: mean 3.2 [SD 1.7] days vs low engagement: mean 2.2 [SD 1.5] days), *t*_32_=1.85, *P*=.07. There were no differences in frequency of other mindfulness practices by the level of text engagement. When mindfulness practice variables were entered simultaneously into a logistic regression analysis predicting abstinence outcomes, mindful awareness of the breath uniquely predicted greater likelihood of abstinence at the end of treatment (β=1.60, *P*=.04) and 1-month follow-up (β=1.99, *P*=.008).

### Associations Between Poverty Status and Abstinence Outcomes by Condition

Among participants living in poverty, 23% (3/13) of those in iQuit Mindfully were abstinent at the end of treatment and 1-month follow-up, whereas none of the participants in the control group receiving MBAT quit smoking at either time point. Fisher’s exact tests examined associations between poverty status and abstinence separately by condition. Poverty status was not significantly associated with abstinence at the end of treatment (MBAT: *P*=.26; iQuit Mindfully: *P*=.67). At 1-month follow-up, living below the poverty level was associated with worse cessation outcomes among MBAT participants (*P*=.03) but not among those receiving iQuit Mindfully (*P*=.65).

## Discussion

### Principal Findings

This pilot study examined the feasibility of a mindfulness-based smoking cessation program incorporating between-session text messaging (*iQuit Mindfully*). To our knowledge, this is the first study to use text messaging to enhance mindfulness-based smoking cessation treatment, and preliminary results support the feasibility and acceptability of text messaging for providing day-to-day smoking cessation support to low-SES, racially/ethnically diverse adults. Strong retention was achieved (76% [54/71] at the end of treatment, and 89% [63/71] at 1-month follow-up); engagement in iQuit Mindfully was high (88% [29/33] indicated reading all or most text messages, and 89% [34/38] texted the system); and participants provided positive ratings and feedback about the text messages. Between-session text messaging could be particularly beneficial for promoting smoking cessation among low-SES adults. Participants provided suggestions for further improving the text messaging program, and the results of this study warrant additional investigation in a larger RCT.

The overall biochemically confirmed smoking cessation rates were 22% (12/55) at the end of treatment and 19% (12/62) at 1-month follow-up, with no differences between conditions. Living below the poverty level predicted worse cessation rates at 1-month follow-up in participants receiving in-person MBAT only, but not among those receiving iQuit Mindfully text messages. Smokers living in poverty not only have lower health care access but also are continually confronted with more tobacco advertising, higher social norms for smoking, and lower social support for quitting and experience higher stress and lower self-efficacy for quitting [3,6,8]. The availability of 24/7 text messaging support could be vital for helping low-SES smokers to overcome these chronic, day-to-day barriers. This study is limited by small sample size, and further investigation in a larger, appropriately powered trial is needed. Extant studies do support the use of mobile phone–delivered interventions for smoking cessation specifically among low-income smokers [50].

### Comparison With Prior Work

Participants who were more engaged with the iQuit Mindfully text messaging program practiced informal mindfulness more frequently and were more likely to quit smoking at the end of treatment. This is consistent with past research, suggesting that higher engagement with mHealth programs predicts better smoking cessation outcomes [51-53]. However, low user engagement is a pervasive problem with mHealth programs [24,54], and efforts are needed to increase engagement with the ultimate goal of improving outcomes. One strategy suggested by our participants is to further personalize text messages, and extant research suggests that tailoring interventions to users’ needs and preferences can indeed increase engagement and efficacy [55-57]. We also examined associations between treatment condition and in-person session attendance. There were no differences in attendance between MBAT and iQuit Mindfully participants, but those who attended more sessions were more likely to quit smoking. Future research might consider how technology could increase in-person session attendance as well as engagement during these sessions.

Greater informal mindfulness practice (ie, mindful attention to breathing throughout the day) predicted higher likelihood of smoking abstinence at both end of treatment and 1-month follow-up. Although more frequent personal mindfulness practice is hypothesized to confer psychosocial benefits, the literature on associations between mindfulness practice and clinical outcomes has been somewhat mixed [58-60]. Past research has shown positive associations between mindfulness practice and better smoking cessation outcomes [11]. The findings of this study suggest that apart from formal meditation practice, mindful attention to breathing in the context of daily activities is uniquely associated with better smoking cessation outcomes. This informal practice (eg, taught through the STOP [*Stop, Take a breath, Observe, Proceed*] acronym in MBAT and other mindfulness programs) could be especially useful for coping with cravings and other stressors during the cessation process.

Overall, iQuit Mindfully participants noted positive experiences with the text messages. Themes included appreciating the positive tone, reminders, mindfulness techniques, social support, timing of the messages, self-compassion in the face of smoking lapses, and coping strategies. This is consistent with our past qualitative work developing iQuit Mindfully [36] as well as other qualitative studies of adults’ experiences with text messaging for smoking cessation [61,62]. A common theme across studies is that although participants understand that the texts are automated, they often describe a sense of social support (eg, “It’s like having a friend who texts you when you are feeling stressed or having a feeling like you want to smoke” [36]). Our participants suggested that the program be even more personalized, and future iterations of the program might provide more flexibility and personalization in terms of frequency, timing, and content of text messages (eg, varying the number and timing of texts based on individual preferences and triggers). As suggested by participants, text messages could also include more religious/spiritual content (this could also be personalized based on individual preferences) and connect them to outside resources (eg, direct connection to quitlines or other support as needed). In addition, the research team noted some logistical issues with participants using their own mobile phones during the study (eg, service interruptions and changing phone numbers), and future studies might offer participants mobile phones with wireless plans to use for the duration of the study. It is possible that these issues may become less common over time, as mobile phone access continues to increase in low-SES populations [63,64].

### Limitations

This pilot study is limited by a small sample size without statistical power to detect group differences in smoking cessation or other outcomes. Although modeling-based approaches can help to address pitfalls of as-treated analyses [65], our sample was too small to fit such models, and results should be viewed as preliminary evidence of feasibility that will need to be tested in larger trials. In addition, iQuit Mindfully text messages were designed to supplement (rather than replace) in-person MBAT sessions, and thus, this program also involves the substantial time and resources associated with in-person treatment. Our decision to include both in-person treatment and text messaging was based on our formative work with low-SES smokers, who noted that text messaging alone (in the absence of other resources such as in-person treatment) would not be sufficient [36]. However, future iterations might consider fully implementing the program through mHealth to increase scalability and reduce costs. In addition, results may or may not generalize to those not included based on the eligibility criteria (eg, people using multiple forms of tobacco, those with psychotic disorders or drug/alcohol abuse). Finally, frequency of between-session mindfulness practice was relatively low in both MBAT and iQuit Mindfully conditions. This is consistent with other research finding that participants often do not practice mindfulness as frequently as directed [12]. Research is needed to examine strategies to promote mindfulness practice among smokers, and as discussed above, more tailored messaging could be one such strategy. Despite limitations, this pilot study is strengthened by the use of biochemical confirmation of smoking behavior; recruitment of predominantly low-SES, racial minority adults; and RCT design, all of which support feasibility for conducting an efficacy RCT.

### Conclusions

Overall, this proof-of-concept study provides strong evidence for feasibility and acceptability of iQuit Mindfully text messages to enhance MBAT by providing between-session support. Offering tailored 24/7 text messaging support could be helpful for low-SES smokers, who have lower access to cessation support and face formidable day-to-day barriers to quitting. Preliminary findings warrant further investigation in an appropriately powered RCT to determine efficacy.

## Abbreviations

CO: carbon monoxide
MBAT: Mindfulness-Based Addiction Treatment
mHealth: mobile health
PHQ: Patient Health Questionnaire
RCT: randomized controlled trial
RR: risk ratio
SES: socioeconomic status

## Appendix

Multimedia Appendix 1 CONSORT‐EHEALTH checklist (V 1.6.1).
